# Habitat use and abundance of an introduced population of the Japanese weasel *(Mustela itatsi)*: Comparison with the native population

**DOI:** 10.1371/journal.pone.0324200

**Published:** 2025-05-30

**Authors:** Kotaro Kubo, Taisei Tachikawa, Miki Hirose, Masami Hasegawa, Eiji Inoue

**Affiliations:** Department of biology, Faculty of science, Toho University, Funabashi, Chiba, Japan; UNAM: Universidad Nacional Autonoma de Mexico, MEXICO

## Abstract

Understanding habitat use and abundance is essential for elucidating the impact of invasive species. Invasive carnivores affect ecosystems by preying on native animals. In Japan, the Japanese weasel (*Mustela itatsi*) is native to the mainland but has been intentionally introduced to many small islands, including Miyakejima Island. We investigated the habitat use and abundance of invasive non-native Japanese weasels on Miyakejima Island via fecal surveys, and for comparison, performed similar surveys for their native conspecifics on Izu-Oshima Island. We constructed a generalized linear mixed model and estimated fecal abundance across the entire island based on the effect of vegetation type on their abundance. On Miyakejima Island, deciduous broadleaf and bamboo forests had positive effects on weasel abundance, whereas grasslands had a negative effect. Conversely, on Izu-Oshima Island, bare ground had a negative effect. Further, the estimated average fecal abundance across Miyakejima and Izu-Ohshima Islands, considering vegetation type, were 7.44 and 4.89 feces samples per km, respectively, suggesting that weasels are well adapted to Miyakejima Island. We also analyzed the fecal DNA of weasels in a specific area on Miyakejima Island and estimated non-native weasel density at 20 individuals per km^2^ (95% CI: 4.9–80) using genetic capture-recapture methods in the area. These findings enhance understanding regarding non-native species and may facilitate the formulation of countermeasures for their control.

## Introduction

The introduction and spread of invasive alien species are now a global concern. Consequently, various countermeasures are under consideration [1]. Carnivores such as raccoons (*Procyon lotor*) and mustelids (*Mustela erminea, M. furo, M. nivalis*), are known to prey on other mammals, amphibians, and reptiles, which negatively impacts ecosystems [2,3]. Weasels and mongooses have been introduced worldwide in attempt to control rodent populations and prevent damage to agricultural crops [1,4,5]. In particular, least weasels (*Mustela nivalis*) have a history of being intentionally introduced to islands, primarily in Europe and elsewhere [4]. Negative impacts on native species such as birds and invertebrates have been reported following these introductions [6]. The significant ecological impact of introduced species highlights the importance of understanding the environmental conditions under which the target species inhabits an area [7,8]. In addition, the effective control of invasive species requires knowledge of their location and population number [9] given that the required management efforts depend, among others, on the local population density of the species.

In Japan, the Japanese weasel (*Mustela itatsi*) is an endemic species, naturally distributed in Honshu, Shikoku, Kyushu, and the surrounding islands. The abundance of native Japanese weasels has been found to vary depending on the environment: 7.6 fecal samples per km were found in a national forest along a valley in Kochi Prefecture [10], while 1–3.7 fecal samples per km were recorded on the walking trails along the Tama River in Tokyo, where the surrounding environment transitions from natural to urban [11]. Similar to the European case, *M. itatsi* was intentionally introduced to a number of non-native islands, with the aim of mitigating damage to agriculture and forestry from rats [12–14]. Apart from the expected rat-control effect, the naturalization of *M. itatsi* on some islands has resulted in severe negative consequences to the local ecosystems, such as predation of native bird and reptile species, including endemic and endangered species [14–18]. Therefore, weasels are listed on the Ministry of the Environment’s List of Invasive Alien Species for the Prevention of Ecosystem Damage as a species requiring urgent and aggressive control measures [19].

**Fig 1:**
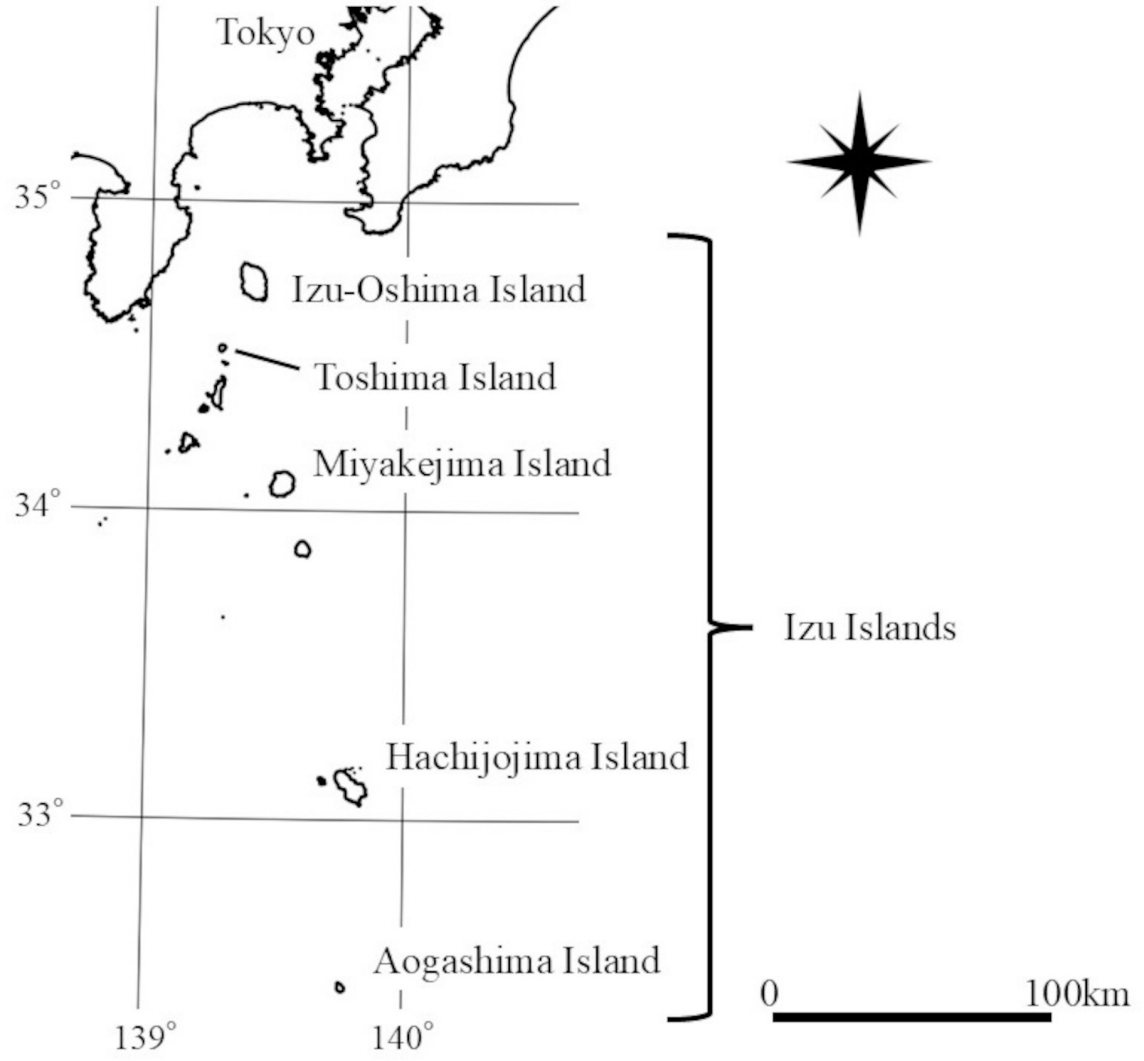
Map of study sites: Miyakejima and Izu-Oshima islands. As for the island names shown on the map, only the islands where the Japanese weasel has been introduced, along with Izu-Oshima Island, which is inhabited by the native Japanese weasel, are displayed. This map was created using digital national land information (National Land Numerical Data (Coastline Data) provided by the Ministry of Land, Infrastructure, Transport and Tourism: https://nlftp.mlit.go.jp/ksj/gml/datalist/KsjTmplt-C23.html).

On the Izu Islands (excluding Izu-Oshima Island), weasels have been intentionally introduced to Toshima Island, Miyakejima Island, Hachijojima Island, and Aogashima Island (Fig 1) [12,20,21]. Although many residing terrestrial vertebrates are not endemic to the Izu Islands, their species composition is unique [22]. Introduced weasels have had a profound impact on ecosystems; for example, the predation of native species, such as the Izu Island thrush (*Turdus celaenops*) and Okada’s blue-tailed skink (*Plestiodon latiscutatus*), has led to significant population decline on Miyakejima Island [12]. Therefore, clarifying the habitat use and density of Japanese weasels is essential for understanding its impacts, and developing future conservation measures for native species on the island.

This study was conducted on the islands of Miyakejima (55 km^2^) and Izu-Oshima (91 km^2^), where the Japanese weasel is introduced and native, respectively [12,20,21]. On Miyakejima Island, only males (20 individuals) were initially released in 1975 as a trial release to assess the invasion risk for native species on this island [16,23]. Despite the reported ecosystem impacts of the initial release, an unofficial release involving females was conducted around 1982 [12,16]. Details such as the number of animals and release location remain unknown due to the unofficial nature of the release [12]. On the Miyakejima and Izu-Oshima Islands, there are no indigenous carnivores (besides the Japanese weasel on Izu-Oshima Island), while wild mice (*Apodemus speciosus* and *Mus musculus*) are known to inhabit both islands [24]. We investigated differences in the introduced Japanese weasel abundance across various vegetation types on Miyakejima Island via fecal surveys, to identify areas on the island that are highly affected by invasive non-native Japanese weasel. Further, as it is challenging to evaluate whether non-native species are well adapted to non-native habitats using only their abundance, we also investigated the abundance of native Japanese weasels on Izu-Oshima Island for comparison. Additionally, we estimated population density, which is a crucial factor for implementing effective control measures. For invasive species, the catch per unit effort (CPUE) has been used as an index of abundance [25,26]. In Japanese weasels, females are more difficult to trap than males [27]. Therefore, CPUE may be an unreliable indicator of weasel abundance because its application to estimate the abundance of females, which are important for reproduction, is unreliable, and the sex ratio of *Mustela* species has reported to be unstable over time [28]. Thus, we employed capture-recapture methods utilizing fecal DNA, a technique that has been applied to various mammals, including carnivores [29,30]. Specifically, we employed this method to estimate the density of weasels in one region on Miyakejima Island.

## Materials and methods

### Study area and field survey

Miyakejima and Izu-Oshima Islands are volcanic islands without rivers. Specifically, on Miyakejima Island, the area around Mt. Oyama in the central part of the island is bare ground, while grasslands, dominated by species such as *Miscanthus condensatus*, extend south of this central region. Further, deciduous broadleaf forests, comprising species such as *Prunus speciosa* and *Alnus sieboldiana*, are located in the upper center region of the island, and evergreen broadleaf forests, comprising species such as *Shiia sieboldii* and *Machilus thunbergii*, are distributed throughout the island. Artificial land, bamboo forests, and fields are located close to the island shore. Tree and vegetation cover in this island have declined considerably ever since the 2000 volcanic eruption [31]. This volcanic eruption resulted in bare areas, where vegetation was lost immediately after the event and has not recovered ever since, and areas where tree cover decreased considerably immediately after the event but have been replaced by grasslands and vegetation is beginning to recover [32].

On Izu-Oshima Island, bare ground and grasslands dominated by silver grasses are located near the summit of Mt. Mihara in the central part of Izu-Oshima Island [33]. Deciduous broadleaf forests comprising species such as *P. speciosa* and *A*. *sieboldiana* are distributed inside and outside the caldera, whereas evergreen broadleaf forests comprising species such as *S. sieboldii* and *M. thunbergii* are distributed throughout the island [33]. Artificial land, bamboo forests, and fields are concentrated in the northern and southern regions of the island. The detailed vegetation maps for both islands are shown in S1 Fig.

**Fig 2:**
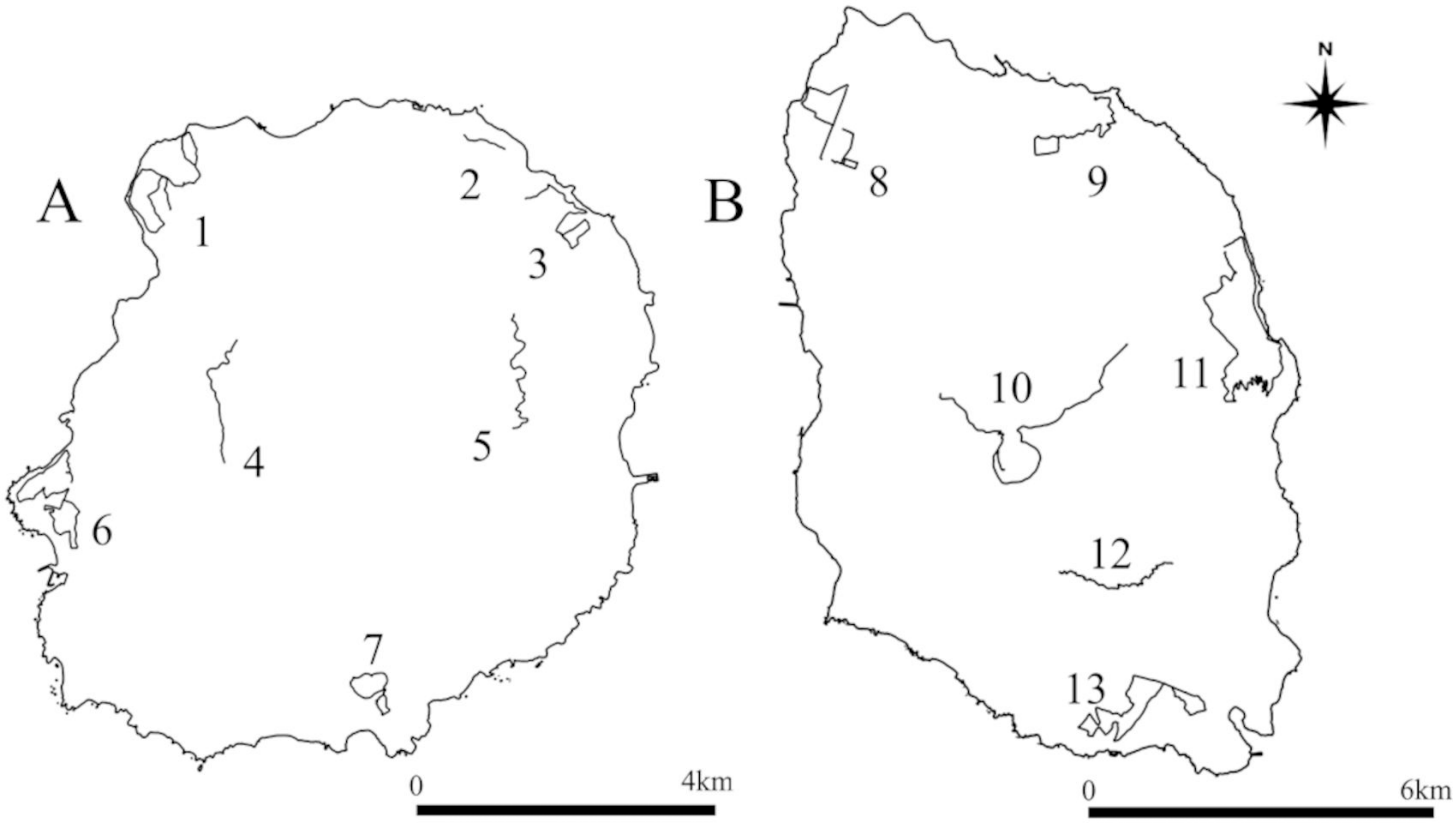
Fecal survey routes on Miyakejima (A) and Izu-Oshima (B) islands. Fecal surveys were conducted on seven routes on Miyakejima island (ID 1–7) and six routes on Izu-Oshima island (ID 8–13). Black lines represent the route paths. Fecal samples were collected on Route ID 1 for genetic density estimation. This map was created using digital national land information (National Land Numerical Data (Coastline Data) provided by the Ministry of Land, Infrastructure, Transport and Tourism: https://nlftp.mlit.go.jp/ksj/gml/datalist/KsjTmplt-C23.html).

For sample collection, we selected seven routes on Miyakejima Island and six routes on Izu-Oshima Island to cover large areas and include various vegetation types on both islands (Fig 2). The routes were established along the relatively narrow roads on the islands that are paved or maintained and are accessible to people and vehicles. Further, the routes were chosen to ensure that the environments surrounding each route was similar. Thus, the lengths of the routes varied depending on the surrounding environments (Table 1). In 2020, fecal surveys were conducted on the Miyakejima Island from March 15–18 and Izu-Oshima Island routes from March 24–26. Two investigators walked on both sides of the road, and recorded their positions upon locating feces using a Garmin GPS unit (GPSMAP 62sc) when feces were found on roads within approximately one meter from the edge. The distance walked and number of feces were used to calculate the number of feces per km (Table 1). Since the survey was conducted in public areas on both islands, no permits were required for entry or research.

**Table 1:** Survey routes and number of feces on Miyakejima and Izu-Oshima islands.

Island	Route ID	Number of feces	Length of route (km)	Number of feces per km
Miyakejima	1	33	5.0	6.6
	2	8	0.6	13.3
	3	13	3.3	3.9
	4	37	2.2	16.5
	5	8	2.5	3.2
	6	14	5.2	2.7
	7	17	2.8	6.0
Izu-Oshima	8	3	5.9	0.5
	9	18	5.6	3.2
	10	10	8.3	1.2
	11	18	10.8	1.7
	12	53	3.2	16.6
	13	17	8.1	2.1

### Estimation of weasel habitat use and feces abundance across both islands

The map of the two islands was divided into 200 m × 200m grid cells, to approximately match the home range size of the weasels (approximately 4.2 ha in male [20]). Land use data were collected from the Japan Aerospace Exploration Agency (JAXA) using high-resolution land use (versions 18.03 and 21.11), at a resolution of 10 m [34]. The area of each of the ten vegetation types (fruit and vegetable fields, artificial land, evergreen coniferous forests, grasslands, bare ground, deciduous broadleaf forests, deciduous coniferous forests, bamboo forests, evergreen broadleaf forests, and rice paddies) in each grid was calculated. In this study, artificial land referred to residential areas and facilities where people are active. Although we categorized fruit and vegetable fields (hereafter referred to as fields) and rice paddies separately, we excluded rice paddies from the analysis, as they were absent within both islands. We conducted a Generalized Linear Mixed Model (GLMM) analysis to examine the effect of environmental factors on Japanese weasel abundance on both islands. In the GLMM, the number of Japanese weasel feces collected from each grid cell was used as the response variable. The area of each vegetation type was included as an explanatory variable, and the length of the survey route within each grid cell was included as the offset term. To examine factors that may have influenced Japanese weasel abundance, we listed models with ΔAIC <2, in addition to models with the smallest AIC. The ‘glmmTMB’ package [35], which can implement a zero-inflated Poisson model which contained a random effect of route IDs, was used for GLMM analyses in R version 4.2.2 [36]. On both islands, the area of deciduous coniferous forests was excluded from the GLMM explanatory variables because it was less than 0.5% of the total area of the islands. Prior to GLMM analysis, the ‘usdm’ R package [37] was used to calculate the variance inflation factor (VIF) for each response variable. For both islands, the area of evergreen broadleaf forests was excluded from the explanatory variables because the VIF values exceeded five.

To compare total weasel abundance on both islands, we calculated the estimated average fecal abundance (i.e., the average number of feces per km across all grid cells) on both islands. The coefficient estimates from the model with the smallest ΔAIC for each island were extrapolated to 200 m × 200 m grid cells across the entire area of both islands to calculate fecal abundance in each grid, and the average value was then calculated for each island.

### DNA sampling and genetic analysis

We collected fecal samples for genetic density estimation of weasels along the route at Izumisaki, Miyakejima Island (Fig 2, Route ID 1) from March 18 to March 20, 2021. Fecal samples were not collected if they appeared to be old. Fecal samples were preserved in lysis buffer (0.5% SDS, 100 mM EDTA pH 8.0, 100 mM Tris-HCl, pH 8.0, and 10 mM NaCl [38]) before DNA extraction. DNA was extracted from feces using the QIAamp DNA Stool Mini Kit (QIAGEN, Tokyo, Japan) and eluted with 200 µl of water.

We used six microsatellite loci: four developed for the European mink (MLUT 32, MLUT 25, MULT20, and MLUT04 [39]), one developed for the Japanese weasel (Mi3007 [40]; direct submission to GenBank, accession no. AB609143), and one developed for the Siberian weasel (Ms65 [41]). Among the six microsatellite loci, Mi3007 includes a tetra-nucleotide repeat motif, while the other loci have di-nucleotide repeat motifs.

Multiplex polymerase chain reaction (PCR) was performed for all six microsatellite loci. The PCR amplification mixture consisted of 2 µl template DNA solution, 5 µl QIAGEN multiplex PCR Master Mix (QIAGEN), and 0.2 µM of each primer (Table 2), for a total volume of 10 µl. After incubating at 95 °C for 15 min, PCR was performed as follows: 45 cycles of denaturation at 94 °C for 30 s, annealing at 57 °C for 30 s and extension at 72 °C for 30 s, followed by a final extension at 60 °C for 30 min. Fragment analysis was performed with Fasmac (Kanagawa, Japan) using an Applied Biosystems 3730xl DNA Analyzer (Thermo Fisher Scientific, Kanagawa, Japan), and genotypes were determined using Peak Scanner Software version 2.0 (Thermo Fisher Scientific). PCR was conducted at least three times, and genotypes were determined when at least two independent PCRs yielded the same genotypes for heterozygotes and at least three independent PCRs yielded the same genotypes for homozygotes [42]. MICRO-CHECKER 2.2 was used to confirm the absence of null alleles in the final dataset [43]. GenAlEx version 6.5 [44] was used to determine the number of alleles at each locus (*Na*), observed (*Ho*) and expected (*He*) heterozygosity, and the probabilities of genotype matching between two individuals (*PI*) and between siblings (*PIsib*).

**Table 2:** Genetic characteristics of the six microsatellite loci used in the Japanese weasels on Izumisaki (Route ID 1), Miyakejima island.

Locus	Label	Range	*N*	*Na*	*Ne*	*Ho*	*He*	*PI*	*PIsib*
Mi3007	FAM	112–128	8	3	1.47	0.38	0.32	0.49	0.71
MLUT32	FAM	144–166	8	2	1.88	0.50	0.47	0.39	0.61
Ms65	FAM	192–200	8	3	2.17	0.63	0.54	0.31	0.56
MLUT25	HEX	112–120	8	3	1.86	0.50	0.46	0.35	0.61
MLUT20	HEX	168–176	8	3	2.61	0.63	0.62	0.22	0.50
MLUT04	NED	98–104	8	3	2.13	0.75	0.53	0.28	0.56

Range, range of allele sizes; *N*, number of samples that yielded genotypes; *Na*, number of alleles; *Ne*, effective number of alleles; *Ho*, observed heterozygosity; *He*, expected heterozygosity; *PI*, probability of genotype matching between two nonrelatives; *PIsib*, probability of genotype matching between siblings.

Although we selected Route IDs 1 and 6 for genetic density estimation considering their accessibility to the survey site, the low success rate of individual identification from fecal DNA and the relatively small sample size (26) prevented us from estimating density for Route ID 6. Thus, we presented only the results for Route ID 1 in the text.

### Estimation of density in Izumisaki (Route ID 1), Miyakejima Island

We genotyped 12 samples and identified eight of the 105 samples collected on Route ID 1 (see Results section). We estimated weasel density using geographical data from 12 samples (eight individuals, see the Results section) obtained via the spatially explicit capture-recapture method (SECR [45]), which offers to estimate population density rather than the number of individuals. The analysis was performed in R version 4.2.2 using the ‘secr’ package version 5.0.0 [46]. We computed the frequency of distances between sampling points where feces from the same Japanese weasel were collected. We set the detector type as ‘count’ and the detection function as ‘half normal’. The buffer width should be long enough to cover the entire range of animal movement [46]. The estimated home range size of Japanese weasels is reported to be approximately 4.2 ha [15], which roughly corresponds to an area of a single 200 m × 200m grid cell. Thus, the buffer width was set to 400 m, i.e., two-fold 200 m. We adopted two models: a null model, in which the capture probability was assumed to be equal among all individuals, and a two-class finite mixture model, in which the capture probability was assumed to be classified into two categories, given that the probability might be biased by sex. We selected an appropriate model using the Akaike Information Criterion (AIC) [47].

## Results

### Fecal survey

The number of feces samples per km along the seven routes on Miyakejima Island ranged from 2.7 to 16.5 (Table 1). On Izu-Oshima Island, the number of feces per km on the six routes ranged from 0.5 to 16.6 (Table 1). On both islands, the number of fecal samples varied between routes, and notably, we obtained low feces numbers for all routes on Izu-Oshima Island except for Route ID 12.

### Factors affecting weasel abundance and abundance estimation across both islands

For Miyakejima Island, the model that included grassland, deciduous broadleaf forest, and bamboo forest yielded the lowest AIC value (AIC = 315.9; Table 3), and based on this model, the coefficient estimate for grassland was negative, whereas those for deciduous broadleaf forest and bamboo forest were positive (Table 3). Grassland and deciduous broadleaf forest were included in almost all models with ΔAIC < 2. For Izu-Oshima Island, the model that included bare ground yielded the lowest AIC value (AIC = 325.8; Table 4), and the coefficient estimate for bare ground was negative. Bare ground was included in all models with ΔAIC < 2 (Table 4).

**Table 3:** Results of GLMM analysis examining the effect of environmental factors on Japanese weasel abundance on Miyakejima Island.

Model	IC	FL	AL	EC	GL	BG	DB	BF	df	AIC	ΔAIC
GL + DB + BF	1.8				−1.8		1.2	4.0	6	315.9	0
GL + DB	1.9				−1.9		1.1		5	316.2	0.3
AL + GL + DB + BF	1.9		−3.1		−1.9		1.3	4.2	7	316.9	1.1
AL + GL + DB	2.0		−2.6		−1.9		1.1		6	317.5	1.7
GL	2.2				−2.0				4	317.6	1.7
GL + BG + DB + BF	1.9				−1.8	−0.3	1.2	3.9	7	317.8	1.9

IC: Intercept; FL: Field; AL: Artificial land; EC: Evergreen coniferous forest; GL: Grassland; BG: Bare ground; DB: Deciduous broadleaf forest; BF: Bamboo forest; df: Degrees of freedom. We listed the model with the lowest AIC as well as models with ΔAIC < 2. The results for all models are shown in S2 Table.

**Table 4:** Results of GLMM analysis examining the effect of environmental factors on Japanese weasel abundance on Izu-Oshima Island.

Model	IC	FL	AL	EC	GL	BG	DB	BF	df	AIC	ΔAIC
BG	1.8					−10.7			4	325.8	0
BG + BF	1.8					−10.2		−6.7	5	326.0	0.2
EC + BG	1.7			0.6		−10.7			5	327.1	1.2
FL + BG + BF	1.8	1.8				−10.2		−8.0	6	327.1	1.3
FL + BG	1.7	1.3				−10.8			5	327.3	1.5
BG + DB	1.8					−10.5	−0.7		5	327.4	1.6
GL + BG	1.7				0.5	−10.6			5	327.5	1.7
BG + DB + BF	1.9					−10.0	−0.7	−6.7	6	327.6	1.7
EC + BG + BF	1.7			0.5		−10.2		−6.0	6	327.6	1.8
GL + BG + BF	1.8				0.5	−10.1		−6.6	6	327.8	1.9

IC: Intercept; FL: Field; AL: Artificial land; EC: Evergreen coniferous forest; GL: Grassland; BG: Bare ground; DB: Deciduous broadleaf forest; BF: Bamboo forest; df: Degrees of freedom. We listed the model with the lowest AIC as well as models with ΔAIC < 2. The results for all models are shown in S3 Table.

**Fig 3:**
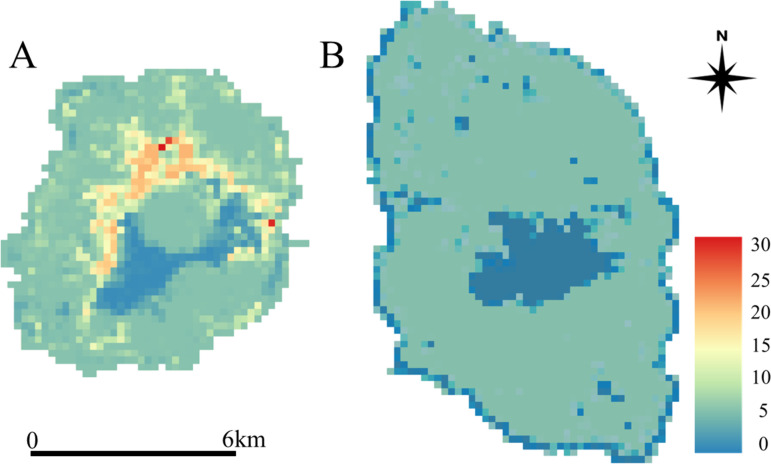
Estimation of Japanese weasel feces per kilometer on Miyakejima Island (A) and Izu-Oshima Island (B). Red represents a higher fecal number per km, blue represents a lower fecal number per km. Data used was provided by JAXA’s High Resolution Land Use and Land Cover Map: https://www.eorc.jaxa.jp/ALOS/jp/dataset/lulc_j.htm.

Using the coefficient estimates from the models with the lowest AIC, we estimated the number of weasel feces per km in each grid cell across both islands (Fig 3). The centers of Izu-Oshima Island were craters classified as bare ground, which resulted in low abundance. The distribution did not vary significantly in other areas of Izu-Oshima Island. However, on Miyakejima Island, the distribution was localized around the northern edge of the central crater. Further, the estimated average fecal abundance across Miyakejima Island was 7.44 feces per km, whereas that across Izu Oshima Island was 4.89 feces per km.

### Individual identification and density estimation in Izumisaki (Route ID 1), Miyakejima Island

We collected 105 fecal samples from Route ID 1. Of the 105 samples, 12 were successfully genotyped for the six microsatellite loci (S1 Table). The genetic diversity of each locus was calculated after excluding samples with identical genotypes. Null alleles were not found at any loci. The number of alleles per locus ranged from 2 to 3, with expected and observed heterozygosity values ranging from 0.32 to 0.62 and from 0.38 to 0.75, respectively (Table 2). The values of *PI* and *PIsib* for the six loci were 9.0 × 10^−4^ and 3.6 × 10^−2^, respectively, indicating high reliability in individual identification.

Of the 12 genotyped samples, we identified 8 individuals (S1 Table). One individual was sampled three times, two were sampled twice, and five were sampled once.

In the SECR analysis, the AIC value of the null model (51.7) was lower than that of the two-class finite-mixture model (55.7). Therefore, the null model was selected. Density estimates by the null model were 20 (95% CI: 4.9–80) individuals per km^2^.

## Discussion

### Environmental factors that affected weasels abundance

Given the intentional introduction of weasels to Miyakejima Island for rat extermination, it is plausible that weasels are found to be abundant in fields. However, artificial land and field were not included in the best model of this study. Even when considering all models with ΔAIC < 2, no model included field and only two models included artificial land, and the coefficients for this variable were negative (Table 3). We set the grid cell size to approximately match the home range size of male Japanese weasels (4.2 ha [15]) and confirmed the continuous distribution of several grid cells including fields. This implies that it was unlikely for weasels feeding in a field in each grid to defecate in another grid with a different vegetation type. Therefore, our results suggest that the effects of artificial land and fields on weasel abundance of Miyakejima Island were likely negligible, or weak to negative if present. The avoidance of agricultural fields has been reported in long-tailed weasels in North America [48]. These are consistent with the result that the highest number of weasel feces was found in mountainous areas (Route ID 4, Table 1) that were far from residential regions. Since the release of Japanese weasels over 40 years ago [49], it is assumed that the species has become widely distributed on Miyakejima Island, reflecting their ecological characteristics.

The effect of bare ground on the distribution of the weasel was negligible on Miyakeiima Island in this study (Table 3), even though bare ground is widely distributed around the crater, and we expected it to have a negative effect on the distribution of the weasels. One possible explanation for this result is that bare ground is distributed not only around the crater but also near the coast of Miyakejima Island. On Hachijojima Island, one of the Izu Islands where weasels were intentionally introduced, marine products (*Ligia hachijoensis*) have been detected in weasel feces [50]. Another previous study reported that Japanese weasels prey on fish in rivers in mainland Japan [51]. During our survey, we recorded several weasel feces in coastal areas on Miyakejima Island. Thus, the use of coastal areas by weasels possibly made the impact of bare ground on their distribution in Miyakejima Island less significant. Other introduced small carnivores, such as the American mink (*Neogale vison*) and the Indian mongoose (*Urva auropunctata*) have been reported to consume fish and littoral animals like crabs [6,52]. Thus, these aquatic animal prey might partially sustain the population of introduced small carnivores.

Grasslands had a negative impact on weasel distribution on Miyakejima Island, while deciduous broadleaf forests had a positive impact (Table 3). These observations may be attributed to differences in food availability. Grasslands on Miyakejima Island are distributed in areas where the soil has been hardened by eruptions and root growth is difficult [53], resulting in fewer insects and other prey for weasels. Further, it has been reported that in deciduous broadleaf forests on Hachijojima Island, weasel feces were abundant and the weasels often feed on insects [50]. Mustelidae species feed on a variety of animals, and their abundance has been reported to be affected by the food resources [54–57]. The introduced American mink has been reported to shift the diet according to the presence of competitors [52]. These implies that food availability possibly affected the abundance of Japanese weasels on Miyakejima Island after they expanding widely, although further analyses are needed to support this conclusion.

Our analysis revealed differences in habitat use patterns for Japanese weasels between Miyakejima and Izu-Oshima Islands. Specifically, on Izu-Oshima Island, bare ground had a negative impact on weasel distribution, whereas its effect was negligible on Miyakejima Island (Tables 3 and 4). As already discussed, the difference in the effect of bare ground can probably be explained by the presence of bare ground along the coast of Miyakejima Island. Moreover, the model for Izu-Oshima Island, which had a low ΔAIC of 0.17, included a negative estimate for bamboo forest, while a positive effect was confirmed on Miyakejima Island. Bamboo forests on Izu-Oshima Island were more concentrated around residential areas, whereas those on Miyakejima Island were located further away from residential areas. Reportedly, weasels prefer diverse landscapes and generally avoid urban areas [58]. These preferences possibly resulted in bamboo forests having different effects on weasel abundance on the two islands. Further ecological studies, considering the diet in each vegetation type on both islands, are required to clarify this difference in detail. Difference in habitat use patterns between native and introduced ranges have also been reported in other Mustelidae species, and it has been pointed out that these differences make it difficult to predict future range expansion of invasive species [1]. Thus, understanding the habitat use patterns of small carnivores in invasive regions is particularly important, especially on islands, which tend to have their specific ecosystem [22].

### Comparison of Japanese weasel abundance between the introduced and native areas

Fecal abundance was lower on all routes on Izu-Oshima Island (except for Route ID 12) than on all routes on Miyakejima Island. The average estimated number of feces samples, considering vegetation types, was also higher on Miyakejima Island (7.44 feces per km) than on Izu-Oshima Island (4.89 feces per km). These results suggested that Japanese weasel abundance was higher in the introduced area than in the native area. The number of feces samples per km in the introduced population was comparable to or higher than that in native mainland Japan (1–3.7 feces samples in the Tama River in Tokyo [11]; 7.6 feces in Nishikuma Valley in Kochi Prefecture [10]). However, our fecal survey was conducted by two persons and survey methods in previous studies were not described in detail. Thus, direct comparison of our results with those reported in the abovementioned previous studies may not be appropriate.

The relatively high fecal abundance on Miyakejima Island suggested that weasels have sufficiently adapted to the environment and have maintained a high population. On Miyakejima Island, weasel population has increased drastically since introduction, possibly owing to their consumption of native lizard species and Okada’s blue-tailed skink, which lack non-native predators [16,22]. Several studies have shown that on Izu Islands, introduced Japanese weasels prey on birds and insects [15,18,50,59]. They are also considered to be opportunistic predators that preys on readily available prey [46]. Other Mustelidae species also feed on a variety of animals, including birds and amphibians [54–57]. In some prey animals, individuals that are naive to predator species have been reported to lack antipredatory behaviors [60,61]. Thus, the Japanese weasels introduced on Miyakejima Island likely consumed various types of animal matter, such as birds and lizards, which might lack antipredatory behaviors, to sustain their own population.

### Management of invasive non-native species

Population density is a crucial parameter that is often taken into consideration during the implementation of effective control measures for invasive species. The estimated density of Japanese weasel in Izumisaki (Route ID 1), Miyakejima Island was 20 individuals per km² (95% CI: 4.9–80). Considering that the average estimated number of feces samples (7.44) was slightly higher than the number found on Route ID 1, the density across Miyakejima Island is likely to be 20 or more individuals per km². However, the width of the 95% confidence interval was large, which was attributed to the low success rate of individual identification from fecal DNA (only 12 of the 105 samples successfully identified individuals). A lower efficiency of DNA extraction from feces has been reported during the wet season than that during the dry season [62]. This study was conducted on islands in the Pacific Ocean where humidity remains high throughout the year. Furthermore, fecal DNA was collected in March, when the relative humidity was as high as 70%, considering the climatological standard normal for the 1991–2020 period on Miyakejima Island [63]. Thus, our study area may have contributed to a low success rate. Density estimation is important for managing invasive species, and therefore methods to enhance the success rate, such as collecting fresh feces or feces during different seasons, are needed to improve the accuracy of density estimation.

The results of this study showed that Japanese weasels are abundant in deciduous broadleaf forests and bamboo forests on Miyakejima Island, and in these areas, these animals have a pronounced effect on prey species. Reportedly, the endangered bird species, the Izu Islands thrush, is affected by introduced weasels, hence the observed drastic decline in their population and reproductive success [18]. The thrushes were reported to be less abundant in deciduous broadleaf forests than in evergreen broadleaf forests on Miyakejima Island [18]. Therefore, the high abundance of weasels in deciduous broadleaf forests might have severe impacts on the population of thrushes in these areas. Additionally, the introduction of the Japanese weasel has also resulted in a rapid decline in the population of Okada’s blue-tailed skinks [12]. Similar to the Japanese weasels, other introduced Mustelidae spices have been reported to affect native ecosystems, and the elimination of the introduced species has led to an increase in native prey species [54,55]. For example, the removal of introduced American mink has led to an increase of common frogs (*Rana temporaria*) and breeding populations of 14 seabird species in the Finnish islands [54,55]. Thus, the elimination of Japanese weasels from Miyakejima Island might lead to an increase in some native species, such as the Izu Islands thrush and Okada’s blue-tailed skink. When considering the control or extermination of Japanese weasels on Miyakejima Island, information regarding their habitat and estimated population, which are revealed by this study, may help to determine the effort required and facilitate the formulation of countermeasures.

## Supporting information

S1 Fig Vegetation maps of Miyakejima (A) and Izu-Ohshima (B) islands. JAXA’s High Resolution Land Use and Land Cover Map was used to illustrate the vegetation on Miyakejima and Izu Oshima. Data used was provided by JAXA’s High Resolution Land Use and Land Cover Map: https://www.eorc.jaxa.jp/ALOS/jp/dataset/lulc_j.htm
(TIF)

S1 Table Genotypes and sampling locations of 12 genotyped fecal samples. Microsatellite genotypes of 8 Japanese weasels at 6 loci (Mi3007, MLUT32, Ms65, MLUT25, MLUT20, and MLUT04) and their sampling locations (UTM, Zone 52N) are presented.
(DOCX)

S2 Table Results for all models from GLMM analysis examining the effect of environmental factors on Japanese weasel abundance on Miyakejima Island. IC: Intercept; FL: Field; AL: Artificial land; EC: Evergreen coniferous forest; GL: Grassland; BG: Bare ground; DB: Deciduous broadleaf forest; BF: Bamboo forest; df: Degrees of freedom.
(DOCX)

S3 Table Results for all models from GLMM analysis examining the effect of environmental factors on Japanese weasel abundance on Izu-Ohshima Island. IC: Intercept; FL: Field; AL: Artificial land; EC: Evergreen coniferous forest; GL: Grassland; BG: Bare ground; DB: Deciduous broadleaf forest; BF: Bamboo forest; df: Degrees of freedom.
(DOCX)
