# Supplementary figures and images for: Habitat use and abundance of an introduced population of the Japanese weasel (Mustela itatsi): Comparison with the native population
Source: PLoS One. 2025 May 30;20(5):e0324200. doi: 10.1371/journal.pone.0324200 (PMC12124565; doi:10.1371/journal.pone.0324200)

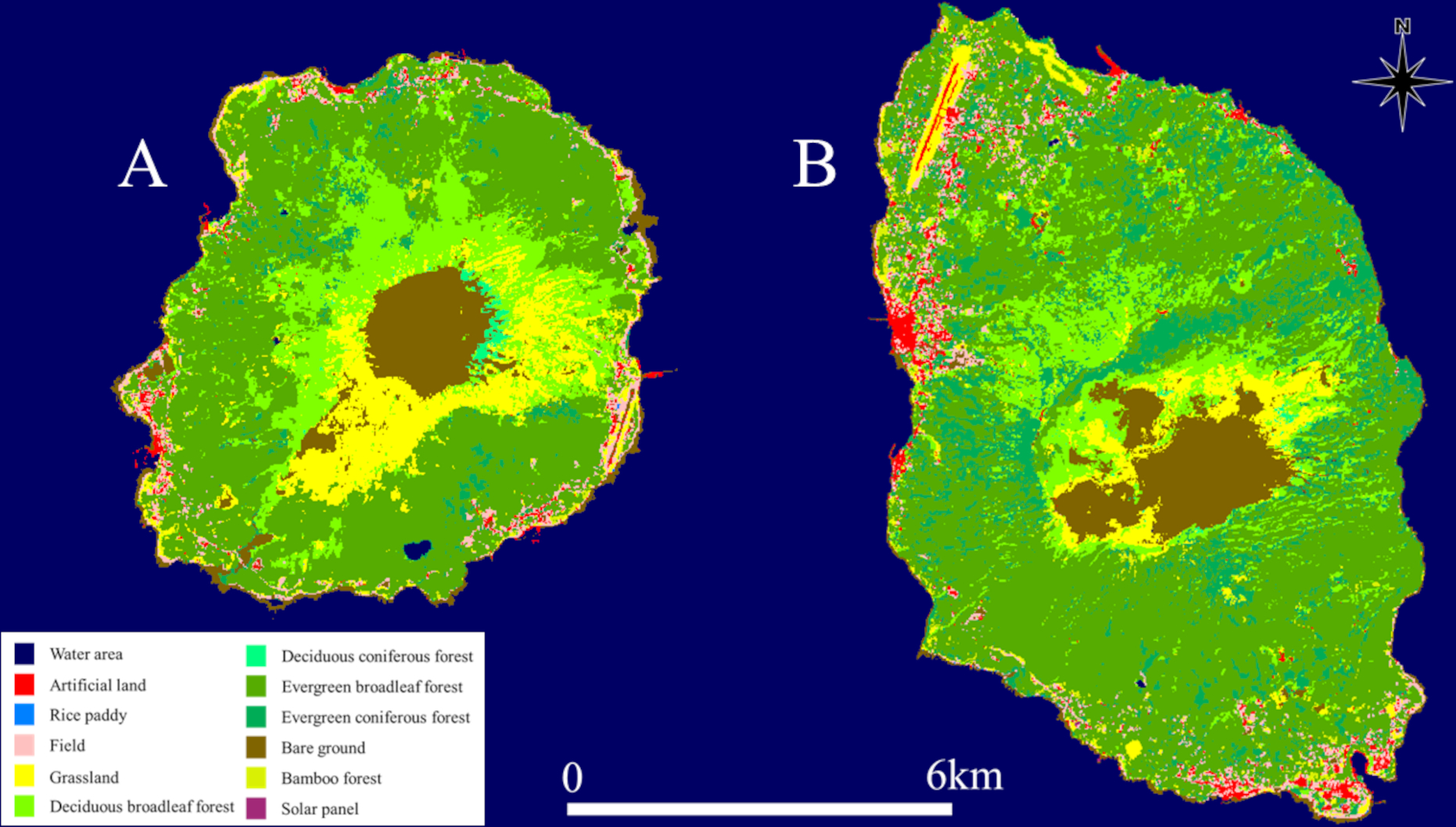

Supplement: S1 Fig — Data used was provided by JAXA’s High Resolution Land Use and Land Cover Map: https://www.eorc.jaxa.jp/ALOS/jp/dataset/lulc_j.htm (TIF) [file pone.0324200.s001.tif]
