# Supplementary material for: Habitat use and abundance of an introduced population of the Japanese weasel (Mustela itatsi): Comparison with the native population
Source: PLoS One. 2025 May 30;20(5):e0324200. doi: 10.1371/journal.pone.0324200 (PMC12124565; doi:10.1371/journal.pone.0324200)
Supplement: S1 Table — Microsatellite genotypes of 8 Japanese weasels at 6 loci (Mi3007, MLUT32, Ms65, MLUT25, MLUT20, and MLUT04) and their sampling locations (UTM, Zone 52N) are presented. (DOCX) [file pone.0324200.s002.docx]

**S1 Table. Genotypes and sampling locations of 12 genotyped fecal samples.**

| Island | Route  ID | Sample　ID | X | Y | Individual  ID | Mi3007 | MLUT32 | Ms65 | MLUT25 | MLUT20 | MLUT04 |
| --- | --- | --- | --- | --- | --- | --- | --- | --- | --- | --- | --- |
| Miyakejima | 1 | 1 | 361060.8 | 3776042 | 1 | 112/120 | 144/166 | 200/200 | 114/120 | 168/168 | 98/98 |
|  |  | 2 | 361085.1 | 3775897 | 1 | 112/120 | 144/166 | 200/200 | 114/120 | 168/168 | 98/98 |
|  |  | 3 | 361239.4 | 3775955 | 1 | 112/120 | 144/166 | 200/200 | 114/120 | 168/168 | 98/98 |
|  |  | 4 | 360826.8 | 3775534 | 2 | 120/120 | 144/144 | 192/192 | 114/120 | 176/176 | 98/102 |
|  |  | 5 | 360899.1 | 3775455 | 2 | 120/120 | 144/144 | 192/192 | 114/120 | 176/176 | 98/102 |
|  |  | 6 | 361157.4 | 3775984 | 3 | 120/120 | 166/166 | 192/200 | 112/120 | 168/170 | 98/102 |
|  |  | 7 | 361029 | 3776071 | 3 | 120/120 | 166/166 | 192/200 | 112/120 | 168/170 | 98/102 |
|  |  | 8 | 360942.3 | 3775381 | 4 | 120/120 | 144/144 | 192/192 | 114/114 | 168/168 | 98/98 |
|  |  | 9 | 361272 | 3775587 | 5 | 120/128 | 144/144 | 192/200 | 114/114 | 170/176 | 98/104 |
|  |  | 10 | 361079.6 | 3776032 | 6 | 120/120 | 144/166 | 192/200 | 114/120 | 168/170 | 98/102 |
|  |  | 11 | 360879.5 | 3776029 | 7 | 120/120 | 144/166 | 192/200 | 114/114 | 168/176 | 98/102 |
|  |  | 12 | 360871.8 | 3775482 | 8 | 112/120 | 144/166 | 192/192 | 114/114 | 168/176 | 98/104 |

Microsatellite genotypes of 8 Japanese weasels at 6 loci (Mi3007, MLUT32, Ms65, MLUT25, MLUT20, and MLUT04) and their sampling locations (UTM, Zone 52N) are presented.
