# Supplementary material for: Habitat use and abundance of an introduced population of the Japanese weasel (Mustela itatsi): Comparison with the native population
Source: PLoS One. 2025 May 30;20(5):e0324200. doi: 10.1371/journal.pone.0324200 (PMC12124565; doi:10.1371/journal.pone.0324200)
Supplement: S2 Table — IC: Intercept; FL: Field; AL: Artificial land; EC: Evergreen coniferous forest; GL: Grassland; BG: Bare ground; DB: Deciduous broadleaf forest; BF: Bamboo forest; df: Degrees of freedom. (DOCX) [file pone.0324200.s003.docx]

**S2 Table.** **Results for all models from GLMM analysis examining the effect of environmental factors on Japanese weasel abundance on Miyakejima Island.**

| Model | IC | FL | AL | EC | GL | BG | DB | BF | df | AIC | ΔAIC |
| --- | --- | --- | --- | --- | --- | --- | --- | --- | --- | --- | --- |
| GL+DB+BF | 1.8 |  |  |  | -1.8 |  | 1.2 | 4.0 | 6 | 315.9 | 0 |
| GL+DB | 1.9 |  |  |  | -1.9 |  | 1.1 |  | 5 | 316.2 | 0.3 |
| AL+GL+DB+BF | 1.9 |  | -3.1 |  | -1.9 |  | 1.3 | 4.3 | 7 | 316.9 | 1.07 |
| AL+GL+DB | 2.0 |  | -2.6 |  | -1.9 |  | 1.1 |  | 6 | 317.5 | 1.65 |
| GL | 2.2 |  |  |  | -2.0 |  |  |  | 4 | 317.6 | 1.74 |
| GL+BG+DB+BF | 1.9 |  |  |  | -1.8 | -0.3 | 1.2 | 3.9 | 7 | 317.8 | 1.91 |
| EC+GL+DB+BF | 1.9 |  |  | -0.3 | -1.9 |  | 1.2 | 4.0 | 7 | 317.8 | 1.96 |
| FL+GL+DB+BF | 1.8 | -0.1 |  |  | -1.8 |  | 1.2 | 4.0 | 7 | 317.9 | 2 |
| GL+BG+DB | 2.0 |  |  |  | -1.9 | -0.4 | 1.1 |  | 6 | 317.9 | 2.05 |
| EC+GL+DB | 2.0 |  |  | -0.4 | -1.9 |  | 1.1 |  | 6 | 318 | 2.19 |
| FL+GL+DB | 1.9 | 0.0 |  |  | -1.9 |  | 1.1 |  | 6 | 318.2 | 2.3 |
| GL+BF | 2.1 |  |  |  | -2.0 |  |  | 3.1 | 5 | 318.2 | 2.38 |
| DB+BF | 1.7 |  |  |  |  |  | 1.2 | 4.1 | 5 | 318.3 | 2.44 |
| DB | 1.8 |  |  |  |  |  | 1.1 |  | 4 | 318.6 | 2.73 |
| AL+GL+BG+DB+BF | 1.9 |  | -3.2 |  | -1.9 | -0.4 | 1.3 | 4.1 | 8 | 318.7 | 2.87 |
| AL+EC+GL+DB+BF | 1.9 |  | -3.2 | -0.4 | -1.9 |  | 1.3 | 4.2 | 8 | 318.8 | 2.96 |
| FL+AL+GL+DB+BF | 1.9 | 0.4 | -3.5 |  | -1.9 |  | 1.3 | 4.2 | 8 | 318.9 | 3 |
| AL+GL+BG+DB | 2.0 |  | -2.8 |  | -1.9 | -0.5 | 1.1 |  | 7 | 319.1 | 3.26 |
| AL+EC+GL+DB | 2.0 |  | -2.8 | -0.5 | -1.9 |  | 1.1 |  | 7 | 319.3 | 3.45 |
| AL+GL | 2.2 |  | -1.5 |  | -2.1 |  |  |  | 5 | 319.4 | 3.54 |
| GL+BG | 2.2 |  |  |  | -2.0 | -0.4 |  |  | 5 | 319.4 | 3.54 |
| FL+AL+GL+DB | 2.0 | 0.4 | -3.0 |  | -1.9 |  | 1.2 |  | 7 | 319.4 | 3.57 |
| EC+GL | 2.2 |  |  | -0.4 | -2.1 |  |  |  | 5 | 319.5 | 3.66 |
| FL+GL | 2.2 | 0.1 |  |  | -2.1 |  |  |  | 5 | 319.6 | 3.73 |
| EC+GL+BG+DB+BF | 1.9 |  |  | -0.3 | -1.9 | -0.3 | 1.2 | 3.8 | 8 | 319.7 | 3.86 |
| FL+GL+BG+DB+BF | 1.8 | 0.1 |  |  | -1.9 | -0.3 | 1.2 | 3.9 | 8 | 319.8 | 3.9 |
| EC+GL+BG+DB | 2.0 |  |  | -0.4 | -1.9 | -0.4 | 1.1 |  | 7 | 319.8 | 3.93 |
| FL+EC+GL+DB+BF | 1.9 | -0.1 |  | -0.3 | -1.9 |  | 1.2 | 4.0 | 8 | 319.8 | 3.95 |
| FL+GL+BG+DB | 2.0 | 0.4 |  |  | -1.9 | -0.5 | 1.1 |  | 7 | 319.8 | 3.99 |
| AL+DB+BF | 1.8 |  | -2.0 |  |  |  | 1.2 | 4.1 | 6 | 319.9 | 4.05 |
| FL+EC+GL+DB | 2.0 | -0.1 |  | -0.4 | -1.9 |  | 1.1 |  | 7 | 320 | 4.19 |
| Model | IC | FL | AL | EC | GL | BG | DB | BF | df | AIC | ΔAIC |
| AL+GL+BF | 2.2 |  | -1.4 |  | -2.0 |  |  | 3.1 | 6 | 320.1 | 4.2 |
| EC+GL+BF | 2.2 |  |  | -0.3 | -2.0 |  |  | 3.1 | 6 | 320.2 | 4.32 |
| GL+BG+BF | 2.2 |  |  |  | -2.0 | -0.2 |  | 3.0 | 6 | 320.2 | 4.32 |
| FL+GL+BF | 2.1 | 0.2 |  |  | -2.0 |  |  | 3.2 | 6 | 320.2 | 4.36 |
| BG+DB+BF | 1.7 |  |  |  |  | -0.2 | 1.2 | 4.0 | 6 | 320.3 | 4.41 |
| AL+DB | 1.9 |  | -1.8 |  |  |  | 1.1 |  | 5 | 320.3 | 4.43 |
| FL+DB+BF | 1.7 | 0.0 |  |  |  |  | 1.2 | 4.1 | 6 | 320.3 | 4.43 |
| EC+DB+BF | 1.7 |  |  | 0.0 |  |  | 1.2 | 4.1 | 6 | 320.3 | 4.43 |
| FL+AL+GL+BG+DB+BF | 1.9 | 0.9 | -4.2 |  | -2.0 | -0.7 | 1.3 | 4.0 | 9 | 320.4 | 4.55 |
| BG+DB | 1.8 |  |  |  |  | -0.3 | 1.1 |  | 5 | 320.4 | 4.58 |
| Null | 2.1 |  |  |  |  |  |  |  | 3 | 320.5 | 4.62 |
| EC+DB | 1.8 |  |  | -0.2 |  |  | 1.1 |  | 5 | 320.6 | 4.72 |
| FL+DB | 1.8 | -0.1 |  |  |  |  | 1.1 |  | 5 | 320.6 | 4.73 |
| AL+EC+GL+BG+DB+BF | 1.9 |  | -3.4 | -0.4 | -1.9 | -0.4 | 1.2 | 4.0 | 9 | 320.6 | 4.73 |
| FL+AL+GL+BG+DB | 2.0 | 1.1 | -4.0 |  | -2.0 | -0.9 | 1.2 |  | 8 | 320.6 | 4.76 |
| FL+AL+EC+GL+DB+BF | 1.9 | 0.3 | -3.5 | -0.4 | -2.0 |  | 1.3 | 4.2 | 9 | 320.8 | 4.91 |
| AL+EC+GL+BG+DB | 2.0 |  | -3.0 | -0.6 | -2.0 | -0.6 | 1.1 |  | 8 | 320.9 | 5.02 |
| BF | 2.0 |  |  |  |  |  |  | 3.3 | 4 | 321 | 5.1 |
| AL+GL+BG | 2.3 |  | -1.8 |  | -2.1 | -0.5 |  |  | 6 | 321.1 | 5.25 |
| FL+AL+EC+GL+DB | 2.0 | 0.4 | -3.1 | -0.5 | -2.0 |  | 1.2 |  | 8 | 321.2 | 5.39 |
| AL+EC+GL | 2.3 |  | -1.6 | -0.4 | -2.1 |  |  |  | 6 | 321.3 | 5.45 |
| FL+GL+BG | 2.2 | 0.5 |  |  | -2.1 | -0.5 |  |  | 6 | 321.3 | 5.45 |
| EC+GL+BG | 2.2 |  |  | -0.4 | -2.1 | -0.4 |  |  | 6 | 321.3 | 5.46 |
| FL+AL+GL | 2.2 | 0.3 | -1.7 |  | -2.1 |  |  |  | 6 | 321.4 | 5.51 |
| FL+EC+GL | 2.2 | 0.1 |  | -0.4 | -2.1 |  |  |  | 6 | 321.5 | 5.66 |
| FL+EC+GL+BG+DB+BF | 1.9 | 0.1 |  | -0.3 | -1.9 | -0.3 | 1.2 | 3.8 | 9 | 321.7 | 5.86 |
| FL+EC+GL+BG+DB | 2.0 | 0.3 |  | -0.4 | -1.9 | -0.5 | 1.1 |  | 8 | 321.7 | 5.89 |
| AL+BG+DB+BF | 1.8 |  | -2.1 |  |  | -0.2 | 1.2 | 4.0 | 7 | 321.8 | 5.97 |
| FL+AL+DB+BF | 1.7 | 0.2 | -2.2 |  |  |  | 1.3 | 4.1 | 7 | 321.9 | 6.02 |
| AL+EC+DB+BF | 1.8 |  | -2.0 | -0.1 |  |  | 1.2 | 4.1 | 7 | 321.9 | 6.04 |
| AL+GL+BG+BF | 2.2 |  | -1.7 |  | -2.0 | -0.3 |  | 2.9 | 7 | 321.9 | 6.08 |
| AL+EC+GL+BF | 2.2 |  | -1.5 | -0.4 | -2.0 |  |  | 3.1 | 7 | 322 | 6.12 |
| FL+AL+GL+BF | 2.2 | 0.4 | -1.7 |  | -2.1 |  |  | 3.1 | 7 | 322 | 6.13 |
| AL+BG+DB | 1.9 |  | -2.0 |  |  | -0.4 | 1.1 |  | 6 | 322.1 | 6.2 |
| Model | IC | FL | AL | EC | GL | BG | DB | BF | df | AIC | ΔAIC |
| FL+GL+BG+BF | 2.1 | 0.4 |  |  | -2.1 | -0.3 |  | 3.0 | 7 | 322.1 | 6.25 |
| EC+GL+BG+BF | 2.2 |  |  | -0.3 | -2.0 | -0.2 |  | 3.0 | 7 | 322.1 | 6.26 |
| FL+EC+GL+BF | 2.1 | 0.2 |  | -0.3 | -2.0 |  |  | 3.1 | 7 | 322.2 | 6.31 |
| AL+EC+DB | 1.9 |  | -1.8 | -0.2 |  |  | 1.1 |  | 6 | 322.3 | 6.4 |
| FL+BG+DB+BF | 1.7 | 0.1 |  |  |  | -0.2 | 1.2 | 4.0 | 7 | 322.3 | 6.4 |
| FL+AL+DB | 1.8 | 0.2 | -1.9 |  |  |  | 1.2 |  | 6 | 322.3 | 6.41 |
| EC+BG+DB+BF | 1.7 |  |  | 0.0 |  | -0.2 | 1.2 | 4.0 | 7 | 322.3 | 6.41 |
| FL+EC+DB+BF | 1.7 | 0.0 |  | 0.0 |  |  | 1.2 | 4.1 | 7 | 322.3 | 6.43 |
| BG | 2.1 |  |  |  |  | -0.4 |  |  | 4 | 322.3 | 6.45 |
| FL+AL+EC+GL+BG+DB+BF | 1.9 | 0.9 | -4.3 | -0.4 | -2.0 | -0.7 | 1.3 | 3.9 | 10 | 322.3 | 6.45 |
| AL | 2.1 |  | -1.0 |  |  |  |  |  | 4 | 322.4 | 6.54 |
| FL+BG+DB | 1.8 | 0.2 |  |  |  | -0.4 | 1.1 |  | 6 | 322.4 | 6.56 |
| EC+BG+DB | 1.8 |  |  | -0.2 |  | -0.3 | 1.1 |  | 6 | 322.4 | 6.56 |
| EC | 2.1 |  |  | -0.2 |  |  |  |  | 4 | 322.4 | 6.58 |
| FL+AL+EC+GL+BG+DB | 2.0 | 1.1 | -4.1 | -0.5 | -2.0 | -0.9 | 1.2 |  | 9 | 322.4 | 6.58 |
| FL | 2.1 | -0.2 |  |  |  |  |  |  | 4 | 322.5 | 6.6 |
| FL+EC+DB | 1.8 | -0.1 |  | -0.2 |  |  | 1.1 |  | 6 | 322.6 | 6.71 |
| FL+AL+GL+BG | 2.3 | 0.9 | -2.6 |  | -2.2 | -0.8 |  |  | 7 | 322.8 | 6.96 |
| AL+BF | 2.0 |  | -1.0 |  |  |  |  | 3.3 | 5 | 322.9 | 7.02 |
| BG+BF | 2.0 |  |  |  |  | -0.2 |  | 3.2 | 5 | 322.9 | 7.06 |
| EC+BF | 2.0 |  |  | -0.2 |  |  |  | 3.3 | 5 | 322.9 | 7.08 |
| FL+BF | 2.0 | -0.1 |  |  |  |  |  | 3.3 | 5 | 323 | 7.1 |
| AL+EC+GL+BG | 2.3 |  | -1.9 | -0.4 | -2.1 | -0.5 |  |  | 7 | 323 | 7.14 |
| FL+EC+GL+BG | 2.2 | 0.4 |  | -0.3 | -2.1 | -0.5 |  |  | 7 | 323.2 | 7.39 |
| FL+AL+EC+GL | 2.2 | 0.2 | -1.7 | -0.4 | -2.1 |  |  |  | 7 | 323.3 | 7.42 |
| FL+AL+GL+BG+BF | 2.2 | 0.8 | -2.3 |  | -2.1 | -0.6 |  | 2.9 | 8 | 323.7 | 7.85 |
| FL+AL+BG+DB+BF | 1.8 | 0.6 | -2.6 |  |  | -0.4 | 1.3 | 4.0 | 8 | 323.7 | 7.85 |
| AL+EC+BG+DB+BF | 1.8 |  | -2.1 | -0.1 |  | -0.2 | 1.2 | 4.0 | 8 | 323.8 | 7.97 |
| AL+EC+GL+BG+BF | 2.2 |  | -1.7 | -0.4 | -2.0 | -0.3 |  | 2.9 | 8 | 323.9 | 8 |
| FL+AL+BG+DB | 1.9 | 0.7 | -2.7 |  |  | -0.6 | 1.2 |  | 7 | 323.9 | 8.02 |
| FL+AL+EC+DB+BF | 1.7 | 0.2 | -2.2 | 0.0 |  |  | 1.3 | 4.1 | 8 | 323.9 | 8.02 |
| FL+AL+EC+GL+BF | 2.2 | 0.3 | -1.7 | -0.3 | -2.1 |  |  | 3.1 | 8 | 323.9 | 8.07 |
| AL+EC+BG+DB | 1.9 |  | -2.0 | -0.2 |  | -0.4 | 1.1 |  | 7 | 324 | 8.17 |
| FL+EC+GL+BG+BF | 2.2 | 0.4 |  | -0.3 | -2.1 | -0.3 |  | 3.0 | 8 | 324.1 | 8.2 |
| Model | IC | FL | AL | EC | GL | BG | DB | BF | df | AIC | ΔAIC |
| AL+BG | 2.1 |  | -1.3 |  |  | -0.4 |  |  | 5 | 324.2 | 8.32 |
| FL+AL+EC+DB | 1.9 | 0.2 | -2.0 | -0.2 |  |  | 1.2 |  | 7 | 324.2 | 8.39 |
| FL+EC+BG+DB+BF | 1.7 | 0.1 |  | 0.0 |  | -0.2 | 1.2 | 4.0 | 8 | 324.3 | 8.4 |
| EC+BG | 2.1 |  |  | -0.2 |  | -0.4 |  |  | 5 | 324.3 | 8.42 |
| FL+BG | 2.1 | 0.1 |  |  |  | -0.4 |  |  | 5 | 324.3 | 8.45 |
| AL+EC | 2.1 |  | -1.0 | -0.3 |  |  |  |  | 5 | 324.4 | 8.5 |
| FL+AL | 2.1 | -0.1 | -0.9 |  |  |  |  |  | 5 | 324.4 | 8.53 |
| FL+EC+BG+DB | 1.8 | 0.2 |  | -0.2 |  | -0.4 | 1.1 |  | 7 | 324.4 | 8.55 |
| FL+EC | 2.1 | -0.2 |  | -0.2 |  |  |  |  | 5 | 324.4 | 8.56 |
| FL+AL+EC+GL+BG | 2.3 | 0.8 | -2.6 | -0.4 | -2.2 | -0.8 |  |  | 8 | 324.7 | 8.89 |
| AL+BG+BF | 2.0 |  | -1.1 |  |  | -0.2 |  | 3.2 | 6 | 324.8 | 8.95 |
| AL+EC+BF | 2.0 |  | -1.0 | -0.2 |  |  |  | 3.3 | 6 | 324.9 | 9 |
| FL+AL+BF | 2.0 | 0.0 | -0.9 |  |  |  |  | 3.3 | 6 | 324.9 | 9.02 |
| EC+BG+BF | 2.0 |  |  | -0.2 |  | -0.2 |  | 3.2 | 6 | 324.9 | 9.04 |
| FL+BG+BF | 2.0 | 0.0 |  |  |  | -0.2 |  | 3.2 | 6 | 324.9 | 9.06 |
| FL+EC+BF | 2.0 | -0.1 |  | -0.2 |  |  |  | 3.3 | 6 | 324.9 | 9.07 |
| FL+AL+EC+GL+BG+BF | 2.2 | 0.8 | -2.4 | -0.3 | -2.1 | -0.6 |  | 2.8 | 9 | 325.6 | 9.79 |
| FL+AL+EC+BG+DB+BF | 1.8 | 0.6 | -2.6 | 0.0 |  | -0.4 | 1.3 | 4.0 | 9 | 325.7 | 9.85 |
| FL+AL+EC+BG+DB | 1.9 | 0.7 | -2.7 | -0.2 |  | -0.6 | 1.2 |  | 8 | 325.9 | 10 |
| AL+EC+BG | 2.1 |  | -1.3 | -0.3 |  | -0.4 |  |  | 6 | 326.1 | 10.28 |
| FL+AL+BG | 2.1 | 0.3 | -1.5 |  |  | -0.5 |  |  | 6 | 326.1 | 10.29 |
| FL+EC+BG | 2.1 | 0.0 |  | -0.2 |  | -0.4 |  |  | 6 | 326.3 | 10.42 |
| FL+AL+EC | 2.1 | -0.1 | -1.0 | -0.3 |  |  |  |  | 6 | 326.3 | 10.49 |
| AL+EC+BG+BF | 2.0 |  | -1.2 | -0.2 |  | -0.2 |  | 3.2 | 7 | 326.8 | 10.93 |
| FL+AL+BG+BF | 2.0 | 0.2 | -1.3 |  |  | -0.3 |  | 3.2 | 7 | 326.8 | 10.94 |
| FL+AL+EC+BF | 2.0 | 0.0 | -1.0 | -0.2 |  |  |  | 3.3 | 7 | 326.9 | 11 |
| FL+EC+BG+BF | 2.0 | 0.0 |  | -0.2 |  | -0.2 |  | 3.2 | 7 | 326.9 | 11.04 |
| FL+AL+EC+BG | 2.1 | 0.3 | -1.6 | -0.2 |  | -0.5 |  |  | 7 | 328.1 | 12.25 |
| FL+AL+EC+BG+BF | 2.0 | 0.2 | -1.3 | -0.2 |  | -0.3 |  | 3.2 | 8 | 328.8 | 12.91 |

IC: Intercept; FL: Field; AL: Artificial land; EC: Evergreen coniferous forest; GL: Grassland; BG: Bare ground; DB: Deciduous broadleaf forest; BF: Bamboo forest; df: Degrees of freedom.
