# Supplementary material for: Habitat use and abundance of an introduced population of the Japanese weasel (Mustela itatsi): Comparison with the native population
Source: PLoS One. 2025 May 30;20(5):e0324200. doi: 10.1371/journal.pone.0324200 (PMC12124565; doi:10.1371/journal.pone.0324200)
Supplement: S3 Table — IC: Intercept; FL: Field; AL: Artificial land; EC: Evergreen coniferous forest; GL: Grassland; BG: Bare ground; DB: Deciduous broadleaf forest; BF: Bamboo forest; df: Degrees of freedom. (DOCX) [file pone.0324200.s004.docx]

**S3 Table.** **Results for all models from GLMM analysis examining the effect of environmental factors on Japanese weasel abundance on Izu-Ohshima Island.**

| Model | IC | FL | AL | EC | GL | BG | DB | BF | df | AIC | ΔAIC |
| --- | --- | --- | --- | --- | --- | --- | --- | --- | --- | --- | --- |
| BG | 1.8 |  |  |  |  | -10.7 |  |  | 4 | 325.8 | 0 |
| BG+BF | 1.8 |  |  |  |  | -10.2 |  | -6.7 | 5 | 326 | 0.17 |
| EC+BG | 1.7 |  |  | 0.6 |  | -10.7 |  |  | 5 | 327.1 | 1.24 |
| FL+BG+BF | 1.8 | 1.8 |  |  |  | -10.2 |  | -8.0 | 6 | 327.1 | 1.28 |
| FL+BG | 1.7 | 1.3 |  |  |  | -10.8 |  |  | 5 | 327.3 | 1.46 |
| BG+DB | 1.8 |  |  |  |  | -10.5 | -0.7 |  | 5 | 327.4 | 1.57 |
| GL+BG | 1.7 |  |  |  | 0.5 | -10.6 |  |  | 5 | 327.5 | 1.69 |
| BG+DB+BF | 1.9 |  |  |  |  | -10.0 | -0.7 | -6.7 | 6 | 327.6 | 1.72 |
| EC+BG+BF | 1.7 |  |  | 0.5 |  | -10.2 |  | -5.9 | 6 | 327.6 | 1.76 |
| GL+BG+BF | 1.8 |  |  |  | 0.5 | -10.1 |  | -6.6 | 6 | 327.8 | 1.92 |
| AL+BG | 1.8 |  | 0.0 |  |  | -10.7 |  |  | 5 | 327.8 | 2 |
| AL+BG+BF | 1.8 |  | 0.4 |  |  | -10.2 |  | -6.9 | 6 | 328 | 2.13 |
| FL+EC+BG | 1.6 | 1.6 |  | 0.7 |  | -10.8 |  |  | 6 | 328.3 | 2.47 |
| FL+EC+BG+BF | 1.7 | 1.9 |  | 0.6 |  | -10.3 |  | -6.9 | 7 | 328.6 | 2.73 |
| EC+GL+BG | 1.6 |  |  | 0.7 | 0.7 | -10.6 |  |  | 6 | 328.6 | 2.77 |
| FL+GL+BG+BF | 1.7 | 1.9 |  |  | 0.6 | -10.1 |  | -7.8 | 7 | 328.8 | 2.94 |
| FL+BG+DB+BF | 1.8 | 1.7 |  |  |  | -10.1 | -0.6 | -7.8 | 7 | 328.8 | 2.95 |
| FL+AL+BG+BF | 1.8 | 2.7 | -1.3 |  |  | -9.9 |  | -7.8 | 7 | 328.8 | 2.99 |
| EC+BG+DB | 1.7 |  |  | 0.5 |  | -10.5 | -0.5 |  | 6 | 328.9 | 3.05 |
| FL+AL+BG | 1.7 | 2.3 | -1.5 |  |  | -10.4 |  |  | 6 | 328.9 | 3.06 |
| FL+GL+BG | 1.7 | 1.4 |  |  | 0.6 | -10.6 |  |  | 6 | 328.9 | 3.07 |
| FL+BG+DB | 1.8 | 1.2 |  |  |  | -10.6 | -0.6 |  | 6 | 328.9 | 3.1 |
| AL+EC+BG | 1.7 |  | 0.2 | 0.7 |  | -10.7 |  |  | 6 | 329.1 | 3.23 |
| EC+GL+BG+BF | 1.7 |  |  | 0.6 | 0.6 | -10.2 |  | -5.7 | 7 | 329.2 | 3.39 |
| GL+BG+DB | 1.8 |  |  |  | 0.4 | -10.4 | -0.6 |  | 6 | 329.3 | 3.41 |
| EC+BG+DB+BF | 1.8 |  |  | 0.4 |  | -10.1 | -0.6 | -6.1 | 7 | 329.3 | 3.5 |
| AL+BG+DB | 1.8 |  | 0.0 |  |  | -10.4 | -0.7 |  | 6 | 329.4 | 3.57 |
| GL+BG+DB+BF | 1.8 |  |  |  | 0.3 | -10.0 | -0.6 | -6.6 | 7 | 329.4 | 3.6 |
| AL+BG+DB+BF | 1.9 |  | 0.3 |  |  | -10.1 | -0.7 | -6.9 | 7 | 329.5 | 3.68 |
| AL+GL+BG | 1.7 |  | 0.0 |  | 0.5 | -10.6 |  |  | 6 | 329.5 | 3.69 |
| AL+EC+BG+BF | 1.7 |  | 0.5 | 0.5 |  | -10.3 |  | -6.1 | 7 | 329.5 | 3.69 |
| Model | IC | FL | AL | EC | GL | BG | DB | BF | df | AIC | ΔAIC |
| FL+EC+GL+BG | 1.5 | 1.7 |  | 0.8 | 0.8 | -10.6 |  |  | 7 | 329.7 | 3.86 |
| AL+GL+BG+BF | 1.8 |  | 0.4 |  | 0.5 | -10.2 |  | -6.8 | 7 | 329.7 | 3.87 |
| FL+AL+EC+BG | 1.6 | 2.5 | -1.3 | 0.7 |  | -10.5 |  |  | 7 | 330 | 4.15 |
| FL+EC+GL+BG+BF | 1.6 | 2.1 |  | 0.7 | 0.7 | -10.2 |  | -6.6 | 8 | 330.1 | 4.22 |
| FL+EC+BG+DB | 1.6 | 1.5 |  | 0.7 |  | -10.7 | -0.4 |  | 7 | 330.2 | 4.37 |
| FL+AL+EC+BG+BF | 1.7 | 2.9 | -1.2 | 0.6 |  | -10.1 |  | -6.9 | 8 | 330.3 | 4.46 |
| FL+EC+BG+DB+BF | 1.7 | 1.8 |  | 0.5 |  | -10.2 | -0.4 | -7.0 | 8 | 330.4 | 4.59 |
| FL+AL+GL+BG+BF | 1.7 | 2.8 | -1.3 |  | 0.6 | -9.8 |  | -7.6 | 8 | 330.5 | 4.64 |
| FL+AL+GL+BG | 1.7 | 2.4 | -1.5 |  | 0.6 | -10.2 |  |  | 7 | 330.5 | 4.67 |
| FL+AL+BG+DB+BF | 1.8 | 2.5 | -1.2 |  |  | -9.9 | -0.6 | -7.6 | 8 | 330.5 | 4.7 |
| EC+GL+BG+DB | 1.6 |  |  | 0.7 | 0.6 | -10.5 | -0.3 |  | 7 | 330.6 | 4.72 |
| AL+EC+GL+BG | 1.6 |  | 0.3 | 0.7 | 0.7 | -10.6 |  |  | 7 | 330.6 | 4.74 |
| FL+AL+BG+DB | 1.8 | 2.2 | -1.4 |  |  | -10.2 | -0.6 |  | 7 | 330.6 | 4.75 |
| FL+GL+BG+DB+BF | 1.7 | 1.7 |  |  | 0.4 | -10.0 | -0.5 | -7.7 | 8 | 330.6 | 4.76 |
| FL+GL+BG+DB | 1.7 | 1.3 |  |  | 0.5 | -10.5 | -0.5 |  | 7 | 330.7 | 4.88 |
| AL+EC+BG+DB | 1.7 |  | 0.2 | 0.6 |  | -10.6 | -0.5 |  | 7 | 330.9 | 5.04 |
| EC+GL+BG+DB+BF | 1.7 |  |  | 0.5 | 0.5 | -10.1 | -0.4 | -5.8 | 8 | 331.1 | 5.28 |
| AL+EC+GL+BG+BF | 1.6 |  | 0.5 | 0.6 | 0.6 | -10.3 |  | -5.9 | 8 | 331.1 | 5.3 |
| AL+GL+BG+DB | 1.8 |  | 0.0 |  | 0.4 | -10.4 | -0.6 |  | 7 | 331.3 | 5.41 |
| AL+EC+BG+DB+BF | 1.8 |  | 0.4 | 0.4 |  | -10.2 | -0.5 | -6.3 | 8 | 331.3 | 5.45 |
| FL+AL+EC+GL+BG | 1.5 | 2.6 | -1.3 | 0.8 | 0.8 | -10.3 |  |  | 8 | 331.4 | 5.55 |
| AL+GL+BG+DB+BF | 1.8 |  | 0.3 |  | 0.3 | -10.1 | -0.6 | -6.8 | 8 | 331.4 | 5.57 |
| FL+EC+GL+BG+DB | 1.5 | 1.7 |  | 0.8 | 0.7 | -10.6 | 0.0 |  | 8 | 331.7 | 5.86 |
| FL+AL+EC+GL+BG+BF | 1.6 | 2.9 | -1.2 | 0.6 | 0.7 | -10.0 |  | -6.5 | 9 | 331.8 | 5.96 |
| FL+AL+EC+BG+DB | 1.6 | 2.4 | -1.3 | 0.6 |  | -10.4 | -0.3 |  | 8 | 331.9 | 6.06 |
| FL+EC+GL+BG+DB+BF | 1.6 | 2.0 |  | 0.6 | 0.6 | -10.2 | -0.1 | -6.7 | 9 | 332.1 | 6.21 |
| FL+AL+EC+BG+DB+BF | 1.7 | 2.7 | -1.2 | 0.5 |  | -10.0 | -0.4 | -6.9 | 9 | 332.2 | 6.35 |
| FL+AL+GL+BG+DB+BF | 1.7 | 2.6 | -1.2 |  | 0.4 | -9.8 | -0.4 | -7.5 | 9 | 332.3 | 6.5 |
| FL+AL+GL+BG+DB | 1.7 | 2.3 | -1.4 |  | 0.5 | -10.2 | -0.4 |  | 8 | 332.4 | 6.52 |
| AL+EC+GL+BG+DB | 1.6 |  | 0.3 | 0.7 | 0.6 | -10.6 | -0.2 |  | 8 | 332.5 | 6.69 |
| AL+EC+GL+BG+DB+BF | 1.7 |  | 0.5 | 0.5 | 0.5 | -10.2 | -0.4 | -6.0 | 9 | 333.1 | 7.21 |
| FL+AL+EC+GL+BG+DB | 1.5 | 2.6 | -1.3 | 0.8 | 0.8 | -10.3 | 0.0 |  | 9 | 333.4 | 7.55 |
| FL+AL+EC+GL+BG+DB+BF | 1.6 | 2.9 | -1.2 | 0.6 | 0.7 | -9.9 | -0.1 | -6.6 | 10 | 333.8 | 7.96 |
| FL+GL+BF | 1.4 | 2.7 |  |  | 2.1 |  |  | -10.2 | 6 | 345.1 | 19.24 |
| Model | IC | FL | AL | EC | GL | BG | DB | BF | df | AIC | ΔAIC |
| GL+BF | 1.5 |  |  |  | 1.9 |  |  | -7.9 | 5 | 345.1 | 19.26 |
| GL | 1.4 |  |  |  | 1.9 |  |  |  | 4 | 345.8 | 19.98 |
| BF | 1.7 |  |  |  |  |  |  | -8.0 | 4 | 346.2 | 20.34 |
| FL+GL+DB+BF | 1.3 | 3.0 |  |  | 2.2 |  | 1.0 | -11.0 | 7 | 346.2 | 20.36 |
| FL+EC+GL+BF | 1.2 | 2.8 |  | 0.7 | 2.2 |  |  | -8.9 | 7 | 346.2 | 20.38 |
| FL+AL+GL+BF | 1.4 | 4.0 | -1.9 |  | 2.1 |  |  | -9.4 | 7 | 346.3 | 20.45 |
| EC+GL+BF | 1.4 |  |  | 0.6 | 1.9 |  |  | -6.8 | 6 | 346.4 | 20.57 |
| EC+GL | 1.3 |  |  | 0.8 | 2.0 |  |  |  | 5 | 346.4 | 20.59 |
| FL+EC+GL+DB+BF | 1.0 | 3.3 |  | 1.0 | 2.4 |  | 1.5 | -9.3 | 8 | 346.5 | 20.68 |
| GL+DB+BF | 1.4 |  |  |  | 2.0 |  | 0.8 | -8.3 | 6 | 346.6 | 20.72 |
| FL+GL | 1.3 | 1.8 |  |  | 2.0 |  |  |  | 5 | 346.7 | 20.88 |
| FL+BF | 1.6 | 2.3 |  |  |  |  |  | -10.1 | 5 | 346.8 | 20.92 |
| Null | 1.6 |  |  |  |  |  |  |  | 3 | 346.9 | 21.1 |
| FL+EC+GL | 1.1 | 2.2 |  | 1.0 | 2.2 |  |  |  | 6 | 347 | 21.11 |
| AL+GL+BF | 1.5 |  | 0.4 |  | 1.9 |  |  | -8.3 | 6 | 347.1 | 21.21 |
| FL+AL+GL | 1.3 | 3.5 | -2.5 |  | 2.1 |  |  |  | 6 | 347.3 | 21.41 |
| EC+GL+DB+BF | 1.2 |  |  | 0.9 | 2.1 |  | 1.2 | -6.9 | 7 | 347.3 | 21.5 |
| FL+AL+GL+DB+BF | 1.3 | 4.3 | -1.9 |  | 2.3 |  | 1.1 | -10.2 | 8 | 347.4 | 21.51 |
| EC+GL+DB | 1.1 |  |  | 1.1 | 2.2 |  | 1.1 |  | 6 | 347.5 | 21.62 |
| FL+AL+EC+GL+BF | 1.2 | 4.0 | -1.8 | 0.7 | 2.2 |  |  | -8.2 | 8 | 347.5 | 21.66 |
| FL+EC+GL+DB | 0.9 | 2.5 |  | 1.3 | 2.4 |  | 1.4 |  | 7 | 347.5 | 21.68 |
| GL+DB | 1.3 |  |  |  | 1.9 |  | 0.6 |  | 5 | 347.5 | 21.7 |
| FL+AL+EC+GL+DB+BF | 1.0 | 4.5 | -1.9 | 1.0 | 2.5 |  | 1.6 | -8.6 | 9 | 347.7 | 21.88 |
| AL+GL | 1.4 |  | -0.4 |  | 1.9 |  |  |  | 5 | 347.8 | 21.92 |
| FL+AL+EC+GL | 1.2 | 3.6 | -2.3 | 0.9 | 2.2 |  |  |  | 7 | 347.8 | 21.95 |
| EC+BF | 1.6 |  |  | 0.5 |  |  |  | -7.2 | 5 | 347.8 | 21.96 |
| DB+BF | 1.6 |  |  |  |  |  | 0.6 | -8.3 | 5 | 347.9 | 22.02 |
| EC | 1.5 |  |  | 0.7 |  |  |  |  | 4 | 348 | 22.14 |
| AL+BF | 1.7 |  | 0.4 |  |  |  |  | -8.4 | 5 | 348.1 | 22.3 |
| FL+AL+BF | 1.6 | 3.6 | -1.7 |  |  |  |  | -9.6 | 6 | 348.2 | 22.33 |
| FL+AL+EC+GL+DB | 0.9 | 4.1 | -2.4 | 1.2 | 2.5 |  | 1.5 |  | 8 | 348.2 | 22.38 |
| FL+DB+BF | 1.5 | 2.5 |  |  |  |  | 0.8 | -10.6 | 6 | 348.2 | 22.39 |
| FL+EC+BF | 1.5 | 2.4 |  | 0.5 |  |  |  | -9.1 | 6 | 348.3 | 22.42 |
| FL | 1.5 | 1.4 |  |  |  |  |  |  | 4 | 348.3 | 22.44 |
| Model | IC | FL | AL | EC | GL | BG | DB | BF | df | AIC | ΔAIC |
| FL+GL+DB | 1.2 | 2.0 |  |  | 2.1 |  | 0.7 |  | 6 | 348.3 | 22.46 |
| AL+EC+GL+BF | 1.3 |  | 0.5 | 0.6 | 1.9 |  |  | -7.2 | 7 | 348.3 | 22.49 |
| AL+EC+GL | 1.3 |  | -0.1 | 0.8 | 2.0 |  |  |  | 6 | 348.4 | 22.58 |
| AL+GL+DB+BF | 1.4 |  | 0.4 |  | 2.0 |  | 0.8 | -8.8 | 7 | 348.5 | 22.66 |
| FL+AL+GL+DB | 1.2 | 3.7 | -2.7 |  | 2.2 |  | 0.8 |  | 7 | 348.7 | 22.84 |
| DB | 1.6 |  |  |  |  |  | 0.4 |  | 4 | 348.8 | 22.93 |
| AL | 1.6 |  | -0.4 |  |  |  |  |  | 4 | 348.9 | 23.02 |
| FL+EC | 1.4 | 1.7 |  | 0.8 |  |  |  |  | 5 | 349.1 | 23.24 |
| FL+AL | 1.6 | 3.0 | -2.3 |  |  |  |  |  | 5 | 349.1 | 23.28 |
| EC+DB+BF | 1.5 |  |  | 0.6 |  |  | 0.8 | -7.2 | 6 | 349.2 | 23.34 |
| AL+EC+GL+DB+BF | 1.2 |  | 0.6 | 0.9 | 2.1 |  | 1.2 | -7.5 | 8 | 349.2 | 23.37 |
| FL+EC+DB+BF | 1.4 | 2.7 |  | 0.8 |  |  | 1.1 | -9.4 | 7 | 349.3 | 23.45 |
| EC+DB | 1.4 |  |  | 0.9 |  |  | 0.8 |  | 5 | 349.4 | 23.58 |
| AL+EC+GL+DB | 1.1 |  | 0.0 | 1.1 | 2.2 |  | 1.1 |  | 7 | 349.5 | 23.62 |
| AL+GL+DB | 1.4 |  | -0.4 |  | 1.9 |  | 0.6 |  | 6 | 349.5 | 23.63 |
| FL+AL+DB+BF | 1.6 | 3.8 | -1.7 |  |  |  | 0.8 | -10.1 | 7 | 349.6 | 23.75 |
| FL+AL+EC+BF | 1.5 | 3.6 | -1.6 | 0.5 |  |  |  | -8.7 | 7 | 349.7 | 23.88 |
| AL+EC+BF | 1.6 |  | 0.5 | 0.5 |  |  |  | -7.6 | 6 | 349.7 | 23.89 |
| AL+DB+BF | 1.6 |  | 0.4 |  |  |  | 0.6 | -8.7 | 6 | 349.8 | 23.96 |
| AL+EC | 1.5 |  | -0.2 | 0.7 |  |  |  |  | 5 | 350 | 24.13 |
| FL+DB | 1.5 | 1.5 |  |  |  |  | 0.5 |  | 5 | 350 | 24.2 |
| FL+AL+EC | 1.4 | 3.1 | -2.1 | 0.7 |  |  |  |  | 6 | 350.2 | 24.31 |
| FL+EC+DB | 1.3 | 1.9 |  | 1.0 |  |  | 1.0 |  | 6 | 350.3 | 24.43 |
| AL+DB | 1.6 |  | -0.4 |  |  |  | 0.4 |  | 5 | 350.7 | 24.85 |
| FL+AL+EC+DB+BF | 1.4 | 4.0 | -1.7 | 0.7 |  |  | 1.1 | -8.9 | 8 | 350.7 | 24.85 |
| FL+AL+DB | 1.5 | 3.2 | -2.4 |  |  |  | 0.6 |  | 6 | 350.8 | 24.94 |
| AL+EC+DB+BF | 1.5 |  | 0.6 | 0.7 |  |  | 0.9 | -7.8 | 7 | 351.1 | 25.23 |
| FL+AL+EC+DB | 1.3 | 3.4 | -2.2 | 0.9 |  |  | 1.0 |  | 7 | 351.2 | 25.4 |
| AL+EC+DB | 1.4 |  | -0.1 | 0.8 |  |  | 0.8 |  | 6 | 351.4 | 25.58 |

IC: Intercept; FL: Field; AL: Artificial land; EC: Evergreen coniferous forest; GL: Grassland; BG: Bare ground; DB: Deciduous broadleaf forest; BF: Bamboo forest; df: Degrees of freedom.
